# Digital Life Balance and Adolescent Flourishing: The Mediating Roles of Life Satisfaction and Self-Esteem

**DOI:** 10.3390/bs16060901

**Published:** 2026-06-02

**Authors:** Beatrice Adriana Balgiu, Ana-Maria Radu

**Affiliations:** 1 Department of Career and Educational Training, National University of Science and Technology Politehnica Bucharest, 313 Splaiul Independenței, 060042 Bucharest, Romania; 2 Department of Computer Science and Information and Communication Technology, “Ștefan cel Mare” National Pedagogical College Bacau, 6, Spiru Haret Street, 600114 Bacau, Romania; anamaria.radu@pedagogicbacau.ro

**Keywords:** adolescents, digital life balance, flourishing, life satisfaction, self-esteem

## Abstract

This study aimed to examine the association between digital life balance and flourishing in a sample of adolescents with a particular focus on the mediating roles of self-esteem and life satisfaction in the relationship between the two variables. A cross-sectional survey was conducted with a sample of 338 Romanian adolescents (mean age = 16.17 years; 66% girls) who completed measures of digital life balance (Digital Life Balance Scale), self-esteem (Rosenberg Self-Esteem Scale), life satisfaction (Satisfaction with Life Scale), and flourishing (Flourishing Scale). Data were analyzed using partial least squares structural equation modeling (PLS-SEM). The results showed that digital life balance was positively associated with flourishing both directly (β = 0.125) and indirectly through life satisfaction and self-esteem (β = 0.309). The total association was also significant (β = 0.434) (all *p* < 0.001). These findings suggest that digital life balance represents an important correlate of flourishing in adolescence.

## 1. Introduction

Digital life balance (DLB) is a relatively recent concept that refers to how people manage their interactions with digital technologies in a manner that supports daily activities without disrupting them (Duradoni et al., 2022). From this point of view, DLB reflects the subjective balance between the online and offline spheres of everyday life. The concept is derived from the notion of work–life balance in organizational psychology which emphasizes the management of multiple roles and responsibilities in life (Greenhaus et al., 2003; Sirgy & Lee, 2018).

Digital life balance notion originates from the Psychology of Harmony and Harmonization (Di Fabio & Tsuda, 2018) in which harmony is viewed as a dynamic process based on the balancing of different elements within an organic whole. Harmony can be analyzed at different levels: intrapsychic (between different parts of the body, mind and heart and different purposes), interindividual and between the individual and the natural world/universe (Di Fabio & Tsuda, 2018). A state of disharmony can arise when the balance between online and offline life is disrupted. Such an imbalance can facilitate problematic patterns of Information and Communication Technology (ICT) use, especially excessive involvement in social media platforms.

Adolescents are one of the groups that interact most frequently with ICT and are exposed to large amounts of digital content (Odgers & Jensen, 2020). In Romania, social networks are the main activity of adolescents, exceeding the European average. They spend 6% more time on social platforms than the average young person in the EU (Eurostat, 2025). This aspect can intensify both positive emotional states and psychological vulnerabilities with direct implications for well-being (Odgers, 2021; J. Chen et al., 2024). Maintaining a balance in digital life is essential for the mental health of adolescents (Berk, 2018). Many studies have demonstrated a positive relationship between DLB and indicators of well-being in adolescents (Tosti et al., 2025) and adults (Duradoni et al., 2022). However, the psychological pathways underlying these associations remain insufficiently understood. Building on this gap, the present study aimed to examine the association between DLB and flourishing (FL) as indicators of optimal psychological functioning. Understanding this kind of association requires attention to internal evaluative processes through which adolescents interpret their life circumstances and develop a sense of personal worth.

To better understand the psychological associations linking DLB to flourishing, the present study draws on Self-Determination Theory (SDT; Deci & Ryan, 2000; Ryan & Deci, 2017). In the SDT conception, well-being depends on the needs for autonomy, competence, and relatedness. Digital environments may both support and frustrate these needs by offering opportunities for connection, self-expression, social comparison, and continuous engagement with online content (Valkenburg et al., 2022; Van de Casteele et al., 2024). We consider that the ability to maintain a balance between online and offline activities may reflect an important form of digital self-regulation associated with more positive self-evaluations and greater satisfaction with life. In this framework, self-esteem and life satisfaction were selected as mediating variables because they capture two essential dimensions of adaptive psychological functioning. Self-esteem reflects perceptions of personal worth, competence, and self-acceptance, whereas life satisfaction reflects cognitive appraisal of overall life circumstances. Both constructs have been consistently associated with need satisfaction within the SDT framework and broader indicators of well-being. Therefore, they may represent theoretically relevant pathways linking digital life balance to flourishing.

### 1.1. Relationship Between Digital Life Balance and Well-Being

Research on balance in different areas of life reveals that individuals who perceive a balanced level between different aspects of their lives tend to report higher levels of self-esteem and life satisfaction (Rohrer et al., 2024). In the digital age, this balance involves regulating interactions between online and offline activities. Conversely, maladaptive patterns of technology use, such as problematic Internet use and social media addiction, are consistently associated with lower levels of well-being (Alotaibi et al., 2022; Duradoni et al., 2022). The disharmonious use of ICT results in a reduction in well-being (Çikrıkci, 2016; Kushlev et al., 2019) extending to depressive outcomes in adolescent samples (Sanders et al., 2000; Wang et al., 2025). In contrast, balanced use of ICT is associated with well-being; thus, Internet addiction has been linked to psychological distress and lower levels of life satisfaction (Szabo et al., 2019). Recent research suggests that dispositional mindfulness may mitigate smartphone addiction through its positive association with digital life balance, showing that promoting DLB could be a potential path towards better development among university students (Aldbyani et al., 2025). Empirical evidence has reported a significant positive association between DLB and flourishing, as well as between DLB and positive affect, and a negative relationship between DLB and negative affect, results observed in the general adult population (Duradoni et al., 2022, Tosti et al., 2026) and in students (Soysal et al., 2024). Other studies have shown that high DLB scores are positively associated with psychological well-being and negatively with variables reflecting maladaptive technology use such as anxiety, addiction, negative emotions, and excessive involvement in social networks (Lima-Costa et al., 2024; Tosti et al., 2026). DLB is negatively correlated with psychological inflexibility (Tosti et al., 2026) indicating that it reflects a dispositional ability to adaptively regulate digital engagement in accordance with the Psychology of Harmony and Harmonization (Di Fabio & Tsuda, 2018). With these insights, we hypothesized the following:

**H1.**  *DLB is positively associated with flourishing.*

### 1.2. Relationship Between Digital Life Balance and Life Satisfaction

Life satisfaction (LS) refers to the overall cognitive evaluation of one’s life (Diener et al., 1985). In the case of adolescents, it should be considered a developmental characteristic that supports their psychological development (Goldbeck et al., 2007; X. Chen et al., 2020). Research on digital behaviors suggests that a moderate and controlled level of technology use reduces psychological tension and favors time spent on offline activities with emotional value (Gui et al., 2017). These experiences are consistently linked to higher levels of life satisfaction, suggesting that maintaining an adequate digital balance may contribute to a positive overall evaluation of one’s life (Malas et al., 2025, 2026).

Empirical studies have found that lower levels of digital life balance are associated with reduced life satisfaction and diminished flourishing (Duradoni et al., 2022). Subsequent studies have identified direct links between DLB and life satisfaction in adolescents (Tosti et al., 2025) and adults (Soysal et al., 2024; Malas et al., 2025; Tosti et al., 2026). In the general adult population, it has been observed that high levels of DLB are associated with increased life satisfaction and lower levels of Internet and social media addiction, especially in terms of avoidance behaviors and problematic use of social media (Malas et al., 2025). Our initial hypothesis was as follows:

**H2.**  *DLB is significantly positively associated with life satisfaction.*

### 1.3. Digital Life Balance and Self-Esteem

Self-esteem is an indicator that reflects the general self-evaluation of one’s personal worth and plays a central role in adolescent psychological development (Rosenberg, 1989). As the authors of SDT have shown, self-esteem is much more stable and positive when people perceive their efficacy in meaningful activities (Ryan & Deci, 2017). Many studies have shown that adolescents who spend an extended time on digital platforms are characterized by a lower level of self-esteem, largely due to exposure to upward social comparison, self-idealized presentations and, consequently, the need to post self-idealizations (Valkenburg et al., 2022). Literature has highlighted the negative impact of social media use on self-esteem by increasing feelings of inadequacy (Purnama et al., 2021), and prolonged exposure to screens has been linked to depressive symptoms that further erode it (Boers et al., 2019). For example, excessive use of smartphones and social media can have negative effects such as loneliness (Lapierre, 2020; O’Day & Heimberg, 2021; Pourasadi et al., 2026), lower academic performance (Lepp et al., 2015; Twenge, 2026) and even depression (Ivanova et al., 2020; Liu et al., 2026). In this context, subjective evaluations of digital experiences are essential. Studies have shown that negative perceptions of social media use, especially when associated with high usage time, correlate with lower levels of well-being, whereas positive and agentic mindsets regarding social media use are associated with lower levels of stress and anxiety (Ernala et al., 2022; Lee & Hancock, 2024).

At the same time, the impact of digital media on self-esteem depends on the quality of the online experience, with adolescents differing in their sensitivity to social feedback (Valkenburg et al., 2022). Adolescents who balance their online and offline lives are less likely to base their self-esteem on unstable forms of online validation, and balanced technology use is associated with more stable self-evaluations and better psychological adjustment (Sezgin & Güler, 2020; C. A. Zhang et al., 2023). Empirical evidence also supports a direct association between digital life balance and adolescent self-esteem. For example, Tosti et al. (2025) reported positive correlations between DLB, and self-esteem as measured by the Rosenberg Self-Esteem Scale, suggesting that a structured and moderate approach to technology use may support the development of positive self-evaluations during adolescence.

**H3.**  *DLB is significantly positively associated with self-esteem.*

### 1.4. Relationship Between Life Satisfaction, Self-Esteem and Well-Being

The relationships between life satisfaction and well-being, on the one hand, and self-esteem and well-being, on the other hand, are well established in the literature through empirical studies (L. Zhang et al., 2020; Jarden et al., 2022; Helliwell et al., 2023).

Life satisfaction, considered the cognitive component of subjective well-being, is not only an indication of the evaluation of the quality of one’s existence but also a foundation for broader forms of psychological flourishing (Diener et al., 2010; M. E. P. Seligman, 2011; Ryan & Deci, 2017). Individuals who report higher satisfaction tend to experience positive emotions more frequently, have stronger perceptions of meaning, and engage in worthwhile activities, all of which contribute to higher levels of flourishing (M. E. P. Seligman, 2011). Self-esteem is another essential psychological resource that supports adaptive functioning (Orth & Robins, 2022). Adolescents with high self-esteem tend to exhibit effective coping strategies, social involvement, and a tendency to pursue meaningful personal goals (Goñi Palacios et al., 2015; Harris & Orth, 2020). Meta-analyses examining the development of self-esteem suggest that adolescence is a particularly important stage for consolidating self-esteem, as its developmental trajectory may temporarily stabilize or fluctuate during this period (Orth et al., 2018). Higher levels of self-esteem have been consistently associated with better psychological adjustment and greater well-being throughout life (Caqueo-Urízar et al., 2022).

**H4.**  *Self-esteem is significantly positively associated with flourishing.*

**H5.**  *Life satisfaction is positively and significantly associated with flourishing.*

### 1.5. The Mediating Role of Self-Esteem and Life Satisfaction in the Relationship Between Digital Life Balance and Flourishing

To our knowledge, no study has directly examined the mediating roles of life satisfaction and self-esteem in the relationship between DLB and flourishing. However, existing research supports the plausibility of these associations. As we have shown, positive associations have been documented between DLB and well-being as well as between LS, SE and well-being (Duradoni et al., 2022; Lima-Costa et al., 2024; Tosti et al., 2025) and both mediators have been linked to flourishing in adolescent populations. The theoretical logic of this mediation is based on SDT. A balanced digital life can support the satisfaction of needs for competence and relatedness, creating conditions for more positive self-evaluations and favorable appraisals of one’s own life. When adolescents feel that their digital and offline lives are integrated, rather than competing, they may be less exposed to frustrating need dynamics, such as social comparison and the replacement of meaningful offline activities (Appel et al., 2020; Coyne et al., 2020). Over time, this can translate into a stronger sense of personal worth and a more positive overall evaluation of one’s life, both of which are well-established antecedents of flourishing (Diener et al., 2010; M. E. P. Seligman, 2011). This is particularly relevant during adolescence, when self-esteem and life satisfaction are still being consolidated and remain sensitive to contextual influences (Orth et al., 2018; Goldbeck et al., 2007). Research on related constructs supports this reasoning. Aldbyani et al. (2025) showed that dispositional mindfulness reduces smartphone addiction in part through its positive association with DLB, suggesting that balanced digital engagement represent an important psychological pathway linking intrepresentsulatory resources with well-being-related outcomes. Although the specific mediation tested in the present study has not been previously examined, the convergence of theoretical reasoning and empirical evidence between related constructs provides a basis for the following hypotheses:

**H6.**  *Life satisfaction is expected to statistically mediate the association between digital life balance and flourishing.*

**H7.**  *Self-esteem is expected to statistically mediate the association between digital life balance and flourishing.*

## 2. Materials and Methods

### 2.1. Participants

Participants were recruited from a public National Pedagogical College located in an urban area in Romania. The sample included students enrolled in grades IX–XII in the classes taught by the collaborating teacher involved in data collection. All students were eligible to participate if they were enrolled in the selected classes and were between 14 and 18 years old. No additional exclusion criteria were applied. The link to the questionnaire was distributed to 400 eligible students in the selected classes. Only students for whom parental consent was obtained were given access to the link. Of these, 338 completed the questionnaire, resulting in a response rate of 84.5%. No questionnaires were excluded due to incomplete responses.

### 2.2. Procedure

Data were collected between 17 October and 14 November 2025, using cross-sectional and conventional sampling methods. All respondents were recruited from the same educational institution. The survey was constructed using the instruments below and was posted online via the Google Forms platform. Previous to the participation, parents or legal guardians received an electronic informed consent form distributed via Google Forms. Prior to completing the questionnaire, students were presented with an electronic consent form and asked to actively indicate their voluntary agreement to participate, selecting the following statement: I agree to participate in this study.

The participating subjects completed the instruments anonymously to control bias methods (Tehseen et al., 2017). The survey was administered during supervised classroom sessions, and participants were instructed to complete the questionnaire only once. Although the questionnaire was anonymous, Google Forms settings were configured to reduce the possibility of repeated access to the questionnaire during the data collection session. This procedure was adopted to minimize duplicate submissions and reduce careless or intentionally invalid responses from the participants. In introducing respondents to the survey completion action, a text was posted that explained the purpose of the study, the fact that it involved no risks, and that there were no rewards for completing it. The average time required to complete the measures was approximately 10–12 min.

### 2.3. Ethical Considerations

The study was conducted in accordance with the ethical principles of the World Medical Association Declaration of Helsinki of 1975, as revised in 2013. Approval for the study was granted by the Departmental Ethics Committee of the National University of Science and Technology Politehnica Bucharest (Reg. No. 173/2 September 2025).

### 2.4. Measures

The Digital Life Balance Scale—DLBS (Duradoni et al., 2022) quantifies the global perception of digital balance using four items, one of which is negatively worded and reverse-coded prior to score computation. Items are rated on a 7-point Likert scale: 1 (completely disagree) and 7 (completely agree). Total scores were calculated by summing item responses, with higher scores indicating better digital life balance. Four represents the minimum achievable score on the scale, while twenty-eight represents the maximum achievable score. An example item is as follows: I currently have a good balance between the time I spend online and the time I have available for offline activities. Although digital life balance is a relatively recent construct, previous studies have reported good psychometric properties across different cultural contexts (Duradoni et al., 2022; Tosti et al., 2025; Malas et al., 2025), supporting its use in adolescent samples. The scale was adapted into Romanian in accordance with the recommendations for cross-cultural adaptation of instruments (Sousa & Rojjanasrirat, 2010). Two Romanian English teachers with experience in academic language independently translated the original English version of the scale in collaboration with a psychologist who acted as a conceptual expert. After comparing the translated versions and resolving minor discrepancies, a unified version in Romanian was obtained. This version was subsequently back-translated into English by a bilingual speaker who was unfamiliar with the original instrument, and no relevant semantic or conceptual discrepancies were identified. To assess comprehensibility, the preliminary version was pre-tested on individuals from the target population, including adolescents, and no difficulties in understanding the wording or content of the items were reported. In this study, confirmatory factor analysis (CFA) demonstrated that the scale had the same unifactorial structure as the original one: χ^2^ = 2.324; df = 2; χ^2^/df = 1.162; CFI = 0.999; TLI = 0.998; RMSEA = 0.022 [0.000–0.113]; SRMR = 0.012, *p* = 0.313.

The Rosenberg Self-Esteem Scale—RSES (Rosenberg, 1989) is a widely used instrument designed to measure global self-esteem across various age groups. It comprises 10 statements rated on a 4-point Likert scale, ranging from 1 (strongly disagree) to 4 (strongly agree). Half of the items are negatively worded and require reverse scoring (e.g., I wish I had more respect for myself). The overall self-esteem score was calculated by summing all item responses, with possible scores ranging from 10 to 40 with higher values indicating a stronger sense of self-worth. In this study, we employed the Romanian-adapted version of this scale (Robu et al., 2015). CFA supported an acceptable good fit of the model: χ^2^ = 47.299; df = 22; χ^2^/df = 2.149; CFI = 0.981; TLI = 0.969; RMSEA = 0.058 [0.035–0.081]; SRMR = 0.038, *p* < 0.001.

The Satisfaction with Life Scale—SWLS (Diener et al., 1985) measures life satisfaction (Diener et al., 1985). The instrument included five items (e.g., In most ways my life is close to my ideal) assessed on a scale of 1 (strongly disagree) to 7 (strongly agree). The total score was calculated by summing the item responses, resulting in possible scores between 5 and 35, with higher scores indicating greater life satisfaction. The version used in this study was validated on various samples of Romanian students (Cazan, 2014), including adolescents (Dimitrova et al., 2016). In the present study, CFA highlights a satisfactory model fit supporting the construct validity of the scale: χ^2^ = 9.273; df = 5; χ^2^/df = 1.854; CFI = 0.994; TLI = 0.990; RMSEA = 0.050 [0.000–0.099]; SRMR = 0.018; *p* < 0.001.

The Flourishing Scale—FS (Diener et al., 2010) measures well-being through eight items evaluated on a continuum from 1—strongly disagree to 7—strongly agree. They include information regarding purpose and meaning, supportive and rewarding relationships, involvement and interest, contribution to the well-being of others, feelings of competence, self-acceptance and optimism, and respect from others. A sample item is as follows: “I lead a purposeful and meaningful life.” Scores were summed up to obtain a total flourishing score ranging from 8 to 56. A high score indicates the development of psychological strength and resources. For descriptive purposes, item-level means and standard deviations were additionally reported to facilitate comparability across instruments with different response formats. The scale was validated in the Romanian population and demonstrated good psychometric properties (Balgiu & Simionescu-Panait, 2024). In this study, CFA indicated acceptable model fit: χ^2^ = 32.067; df = 18; χ^2^/df = 1.781; CFI = 0.986; TLI = 0.979; RMSEA = 0.048 [0.018–0.075]; SRMR = 0.030, *p* = 0.022.

Relevant sociodemographic data collected: 1. age, 2. gender, 3. education, and 4. residential environment.

### 2.5. Statistical Strategies

Descriptive statistics (means, standard deviations, skewness and kurtosis, omega McDonald) were used to capture the characteristics of the analyzed group and to evaluate the normality of the data. Because the analyzed variables did not show deviations from normality, the association between them was examined using Pearson’s correlations. Although the proposed mediation model was theory-driven, the primary objective of the structural analysis was prediction-oriented, namely, to examine the extent to which digital life balance, self-esteem, and life satisfaction account for variance in flourishing. For this reason, a partial least squares structural equation modeling (PLS-SEM) approach was adopted, as it is particularly appropriate when the research emphasis is placed on explained variance, prediction-oriented estimation, and the modeling of latent variable composites rather than on exact global model fit (Henseler, 2021; Hair et al., 2022). In addition, PLS-SEM does not require multivariate normality and is well suited for bootstrapped mediation analyses. The quality and fit of the model were assessed through indicators that targeted reliability (Cronbach’s alpha), convergent validity (Dijkstra–Henseler’s rho—ρA, Jöreskog’s rho—ρc, average variance extracted—AVE), discriminant validity (heterotrait–monotrait ratio—HTMT), and variance inflation factors (VIFs). According to general recommendations, cut-off values for ρA, ρc and Cronbach’s alpha should exceed 0.70 to indicate acceptable reliability (Hair et al., 2022). The AVE should be at least 0.50 to support convergent validity (Henseler, 2021). For discriminant validity, the HTMT values should remain below 0.90 or, more conservatively, below 0.85 (Kline, 2023). In addition, to demonstrate the absence of multicollinearity, the VIF values should be below 3.30 (Kock, 2015). CFAs were conducted separately to verify the factorial structure of the Romanian versions of the instruments prior to structural modeling. These analyses were performed independently from the structural model and were not intended as covariance-based model testing. Standardized z-scores were used exclusively for the correlational analyses to facilitate comparability across measures with different response formats. All structural analyses were conducted using the original scale scores.

The coefficient of determination (R^2^) was used to assess the explanatory power of the structural model by estimating the proportion of variance explained in the endogenous constructs. A value above 0.60 for R^2^ is considered substantial (Hair et al., 2022). The R^2^ values were interpreted in conjunction with the conceptual proximity between the included psychological constructs and the overall theoretical structure of the model. All inferential analyses were performed at a significance level of *p* < 0.05. To assess the statistical significance of the model parameters, a bootstrapping procedure with 5000 re-samples was performed, in accordance with the recommendations of Henseler et al. (2016).

All data analyses were performed using SPSS, Version 24 (IBM Corp. Armonk, NY, USA), ADANCO 2.4.0 (University of Twente, Enschede, The Netherlands). JASP 0.19.1.0 (University of Amsterdam, Amsterdam, The Netherlands) was used for factor analysis of the instruments.

## 3. Results

### 3.1. Sociodemographic Characteristics of the Sample

The study sample consisted of 338 students aged 14–18 years (Mean age = 16.17; *SD* = 1.26). The majority of participants were girls, representing 66% of the total sample (*n* = 223) (Mean age = 16.29, *SD* = 1.25), while boys constituted 34% (*n* = 115) (Mean age = 15.99, *SD* = 1.26). Regarding the residential environment, the students come from both urban and rural areas, which allows for capturing various characteristics of the development context. Concerning the classes they belonged to, 59.2% of the participants were enrolled in grades IX–X and 40.8% in grades XI–XII, with a relatively equal distribution across both high school cycles (Table 1).

### 3.2. Controlling Common Method Bias (CMB)

First, CMB was calculated because the study used a cross-sectional design based on self-reported measures and was conducted in a school context, where response biases influenced by the desire for conformity or social desirability may occur. Two complementary procedures were used. Harman’s single test was conducted using exploratory factor analysis (EFA) without rotation, in which all variables from the study were entered simultaneously. The analysis indicated a factorial solution with four factors with eigenvalues greater than 1 (KMO = 0.929; Bartlett’s test of sphericity = 4676.704; df = 351; *p* < 0.001), which together explained 52.71% of the total variance. The first factor covered 35.8% of the variability, a value below the 50% threshold mentioned in the literature (Fuller et al., 2016), which suggests the absence of a dominant variance indicating a significant procedural bias. Second, a single-latent factor model was tested using confirmatory factor analysis (CFA). The model showed poor fit indices (χ^2^ = 1500.983; df = 324; χ^2^/df = 4.632; CFI = 0.7237; TLI = 0.715; RMSEA = 0.104 [90% 0.098–0.109]; SRMR = 0.089), indicating that the data did not conform to a unidimensional structure. The results suggest that common method bias is not a significant problem in this study.

### 3.3. Descriptive and Correlational Analyses

The descriptive analysis (Table 2) shows relatively high mean levels for both digital life balance (M = 4.648, *SD* = 1.21) and flourishing (M = 5.302, *SD* = 0.97). The item with the highest mean score on the DLB Scale was the following: In general, I believe that my online and offline lives are balanced (M = 4.745, *SD* = 1.61). On the Flourishing Scale, it was as follows: I am competent and capable in the activities that are important to me (M = 5.712, *SD* = 1.22). Self-esteem had a mean of 2.860 (*SD* = 0.60), suggesting a moderate-to high level, characteristic of adolescent samples. The item with the highest mean value was the following: I believe that I have certain good personal qualities (M = 3.28, *SD* = 0.75). The skewness and kurtosis coefficients were within the acceptable ranges for approximately normal distributions. The skewness ranged from −0.267 to −0.704, indicating a slight tendency towards upwardly skewed distributions, but without severe deviations. The kurtosis values were between −0.555 and 0.334, indicating that the distributions were normal (Kim, 2013). Overall, the data met the requirements for conducting Pearson correlational analyses and prediction-oriented structural modeling. Regarding gender differences, the results show that there are no significant differences between boys and girls in any of the variables analyzed: digital life balance, life satisfaction, flourishing and self-esteem (all *p* > 0.05).

Table 3 highlights the significant relationships between the variables investigated. There are positive correlations between digital life balance (DLB) and flourishing (FL) (r = 0.359), on the one hand, and DLB and life satisfaction, on the other hand (r = 0.229).

These correlations show that adolescents who maintain a more balanced ratio of digital to offline activities tend to report higher levels of flourishing and life satisfaction. Self-esteem was significantly associated with flourishing (r = 0.702), life satisfaction (r = 0.626), and digital life balance (r = 0.295) (all *p* < 0.001). The moderate association between DLB and self-esteem suggests that adolescents who report a more harmonious way of managing their digital life also tend to report a more favorable perception of themselves. We can state that H1–H5 are preliminarily confirmed through correlational analysis and tested more rigorously through the structural model.

### 3.4. The Mediating Role of Self-Esteem and Life Satisfaction

To analyze the importance of self-esteem in the relationship between DLB and FL, we built a structural equation model (SEM). The model evaluation indicators (Table 4) show that all four constructs—digital life balance (DLB), flourishing (FL), self-esteem (SE) and life satisfaction (LS)—present adequate values of reliability and convergent validity. The composite values of reliability (ρA, ρC and α) ranged between 0.774 and 0.885, thus demonstrating good internal consistency for each construct. The absence of collinearity in the model was determined by the fact that the VIF values fell within the range of 1.21–2.78. The AVE values ranged between 0.475 and 0.573. The LS and SE exceeded the minimum criterion of 0.50, and the DLB and FL constructs had values slightly lower than the recommended threshold. Although the AVE values for DLB and FS were slightly below the recommended threshold of 0.50, the CR values exceeded 0.70. The convergent validity may still be considered adequate when CR is satisfactory (Hair et al., 2022). The AVE result can be partially explained by the reduced factor loading of an item from the FS (I actively contribute to the happiness and well-being of others). This may reflect a more prosocial and other-oriented dimension of flourishing that may be less salient during adolescence, which is more focused on autonomy and self-definition. Because the scale’s reliability remained satisfactory and the CFA indicated an acceptable model, the item was retained to preserve the conceptual integrity of the scale.

The HTMT values (Table 5) highlighted an adequate separation between constructs. None of the HTMT values exceeded the threshold of 0.85, which supports the idea that the variables include distinct conceptual content. This result confirms that the model satisfactorily differentiated between the variables.

The results of the structural model (Table 6) revealed significant relationships between all the included variables, supporting the proposed theoretical model linking DLB, self-esteem, life satisfaction, and flourishing.

**Figure 1:**
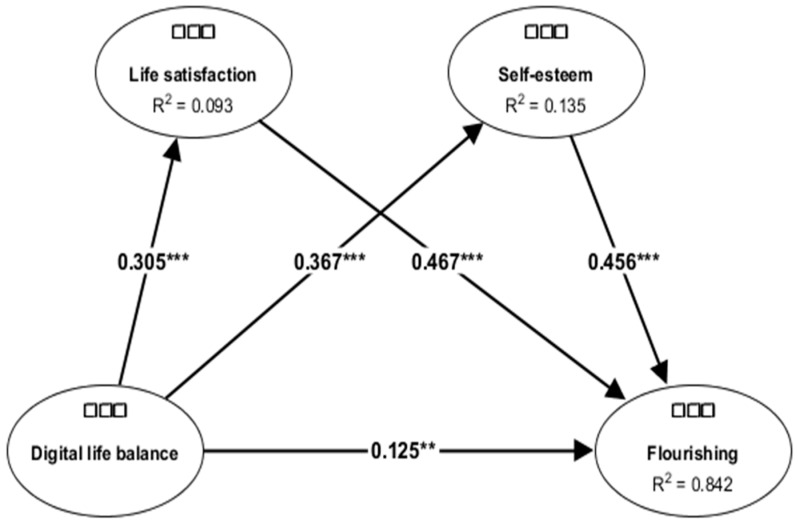
The mediating effect of life satisfaction and self-esteem on the relationship between digital life balance and flourishing (β—path coefficients and R^2^ values). *** *p* < 0.001; ** *p* < 0.01.

Digital life balance had a significant positive direct effect on flourishing (β = 0.125, 95% CI [0.0382, 0.1958]). It was also positively associated with the mediators: self-esteem (β = 0.367, 95% CI [0.2490, 0.4810]) and life satisfaction (β = 0.305, 95% CI [0.1946, 0.4218]). In turn, both self-esteem (β = 0.456, 95% CI [0.3270, 0.5758]) and life satisfaction (β = 0.467, 95% CI [0.3512, 0.5964]) showed significant association with flourishing. The indirect effect of digital life balance on flourishing through life satisfaction and self-esteem was statistically significant (β = 0.309, 95% CI [0.2197, 0.4064]). The total effect was also significant (β = 0.434, 95% CI [0.3236, 0.5405]) (all *p* < 0.001). Both mediators contributed significantly to the relationship between digital life balance and flourishing. The indirect effect through self-esteem was slightly stronger (β = 0.166, 95% CI [0.1036, 0.2490], *p* < 0.001) than the indirect effect through life satisfaction (β = 0.143, 95% CI [0.0857, 0.2160], *p* < 0.001). These results indicate partial mediation because both direct and indirect paths remain significant. Overall, the model suggests that digital life balance is positively associated with adolescent flourishing, both directly and indirectly through life satisfaction and self-esteem. The model explained a substantial proportion of the variance in flourishing (R^2^ = 0.842) indicating strong explanatory power within the present sample. This value, however, should be interpreted with caution given the conceptual proximity of flourishing, life satisfaction and self-esteem (Figure 1). Overall, the model supports positive direct and indirect associations between digital life balance and flourishing through self-esteem and life satisfaction.

## 4. Discussion

This study examined the association between digital life balance and flourishing in adolescents and tested the mediating roles of life satisfaction and self-esteem. The descriptive results showed that adolescents in the sample perceived their digital lives as relatively balanced with the highest scores reflecting a favorable overall appraisal of their digital engagement. These findings are interpreted within the framework of Self-Determination Theory (Ryan & Deci, 2017), which guided the hypothesized pathways linking balanced digital engagement to adolescent flourishing through need-relevant evaluative processes.

The structural equation model showed that digital life balance was positively associated with flourishing, a finding consistent with previous studies linking balanced ICT use to better psychological adaptation and higher levels of flourishing (Duradoni et al., 2022; Soysal et al., 2024; Tosti et al., 2025). In the present study, this relationship remained significant even after including the mediating variables. Thus, it can be seen that the way adolescents regulate their involvement in the digital world has both direct and indirect implications for their psychological functioning. From this point of view, digital life balance may be interpreted as a potential psychological indicator of flourishing in adolescence. These findings also contribute to the emerging validation of DLB as a meaningful construct in adolescent populations. The relationship between digital life balance and life satisfaction was significant but moderate (β = 0.305). Life satisfaction represents a broad cognitive assessment that integrates several domains of life from family to school and social relationships (Tian et al., 2015; Diener et al., 2018). This broader scope may explain why digital experiences constitute only one component of adolescents’ overall life evaluation. In contrast, the association between digital life balance and self-esteem was stronger (β = 0.367), suggesting that the regulation of digital behavior may have more immediate implications for adolescents’ self-evaluation. This finding is consistent with previous research on adolescent samples (Tosti et al., 2025). One possible explanation, consistent with SDT, is that adolescents who achieve a better balance between online and offline activities may experience a greater sense of control over their daily routines and maintain a stronger sense of autonomy in relation to digital environments. These experiences may directly support the development of a more stable and positive self-concept.

Life satisfaction and self-esteem showed strong positive associations with flourishing, confirming their consistently identified roles in adolescent well-being (Orth & Robins, 2022; Parola & Marcionetti, 2023). These findings are consistent with contemporary models of optimal psychological functioning which show that flourishing results from the integration of positive life appraisal and a stable sense of personal worth (Diener et al., 2010; M. Seligman, 2018). From an SDT perspective, both mediators reflect the degree to which fundamental psychological needs are met: life satisfaction captures the extent to which adolescents feel their autonomy and relational needs are fulfilled across life domains, whereas self-esteem reflects the satisfaction of the need for competence. In this sense, well-being results not only from positive experiences but also from the integration of these experiences into a coherent perception of one’s own life and a sense of valued self.

Mediation analysis showed that digital life balance was indirectly associated with flourishing through life satisfaction and self-esteem, consistent with a pattern of partial mediation. Digital life balance may reflect a contextual form of self-regulation, which is positively associated with adolescents’ evaluations of their lives and themselves. Since part of the effect remains direct, we believe that a balanced use of digital technologies can reduce psychological conflicts associated with excessive technology use, such as time spent online competing with school activities (Coyne et al., 2020; Valkenburg et al., 2022) and offline social relationships (Appel et al., 2020)—independently of how adolescents evaluate themselves or their lives.

The large proportion of explained variance for flourishing (R^2^ = 0.842) demonstrates that life satisfaction and self-esteem are important components of positive psychological functioning. This value should be interpreted with caution because the concepts of flourishing and life satisfaction partially overlap in the domain of well-being. It should not be forgotten that the use of self-reported measures may amplify these associations through common method variance. The Flourishing Scale includes items assessing purpose and meaning, positive relationships, self-acceptance, competence, and optimism, which are somewhat related to the core content of the Satisfaction with Life Scale. This conceptual and operational overlap likely contributed to the high variance. Such overlap is not uncommon when multiple constructs related to well-being are included in the same model (e.g., Diener et al., 2010; Huppert & So, 2013). Future studies should examine the distinctiveness of these constructs through more stringent discriminant validity tests (e.g., bifactor modeling) or by using alternative operationalizations of flourishing that minimize content redundancy with life satisfaction.

Considering the cross-sectional design, the directionality of the relationships remains open, and reciprocal bidirectional associations between digital self-regulation and flourishing are plausible. Therefore, the R^2^ value should be interpreted as an indicator of the model’s explanatory power and explained variance within the present sample rather than as definitive evidence of the maximum explanation of adolescent well-being. Further research could test the stability of these associations using longitudinal designs and multi-method approaches—for example, to investigate whether changes in digital life balance over time predict subsequent changes in self-esteem and life satisfaction and whether these changes are associated with flourishing.

Although the present findings showed positive associations between digital life balance and flourishing, previous research on adolescent digital engagement has reported mixed results. Excessive or poorly regulated digital use has been associated with social isolation, problematic social comparison, sleep disruption, depressive symptoms, and lower psychological well-being (Appel et al., 2020; Valkenburg et al., 2022). In this context, the present findings may be explained by the fact that the current study did not examine digital exposure per se, but rather digital life balance, a construct that explicitly captures adolescents’ perceived ability to regulate the integration of online and offline activities in their lives. The results may also be linked to cultural factors, as Romanian adolescents report levels of involvement on social networks above the European average (Eurostat, 2025), while family and school environments may simultaneously encourage interpersonal connectivity and behavioral monitoring, potentially influencing how digital involvement is integrated into life. Cross-cultural comparisons with adolescents from other European and Asian contexts would further clarify the generalizability of these findings.

The study has several limitations that should be considered for an adequate interpretation of the results. The main limitation of this study is related to the composition of the sample, which consisted exclusively of students from one college. This may limit the generalizability of these results. Further studies should use diverse samples from multiple educational institutions or different regions to provide a broader perspective on the phenomenon. Considering that research has documented gender differences in social media use, as well as in sensitivity to online social comparisons (Valkenburg et al., 2022), future studies should ensure a more balanced representation of gender and explicitly examine the potential moderating effects of gender on the proposed model.

Another limitation is the cross-sectional design, which does not allow firm conclusions about the causal direction of the relationships. PLS-SEM analysis revealed significant indirect effects through life satisfaction and self-esteem; however, these findings reflect statistical associations consistent with the hypothesized indirect relationships rather than established causal mechanisms. The directionality of these relationships remains open; it is possible that adolescents with higher levels of flourishing, self-esteem, or life satisfaction may also report more positive perceptions of their digital life balance, suggesting potential bidirectionality. Longitudinal or experimental designs are needed to clarify these causal relationships. Another limitation is the theoretical framework used. Although SDT guided the interpretation of the proposed pathways, the satisfaction of basic psychological needs, autonomy, competence, and relatedness, was not directly assessed.

It should also be noted that DLB was assessed using self-reported scales, which may introduce biases such as subjective perceptions or socially desirable responses. This study used anonymous administration and standardized classroom data collection procedures to reduce socially desirable responses and common method bias. However, the possibility of response bias cannot be completely ruled out. This is especially true for adolescents who may underestimate or overestimate certain aspects of their digital behavior. Future studies should consider combining self-reports with objective measures of technology use. Although preliminary checks did not indicate a single dominant factor, common method variance could not be completely excluded. Subsequent research should address this issue by temporally separating the assessments made or the marker variables to better control method-related variance.

### Theoretical and Practical Implications

At the theoretical level, the present findings extend the literature by revealing the roles of self-esteem and life satisfaction in the association between digital life balance and flourishing. By testing an indirect association model, the study may contribute to a better understanding of the psychological pathways through which balanced ICT use may be linked to adolescents’ well-being. The findings also situate digital life balance within the broader framework of Self-Determination Theory (Ryan & Deci, 2017), suggesting that balanced digital engagement can function as a self-regulatory capacity that supports the satisfaction of fundamental psychological needs—for competence, autonomy, and relatedness—thus creating conditions for flourishing. This may open new directions of research on digital well-being in developing populations.

At the practical level, our results extend the research in the field of education. Starting in 2025, the issue of rigorous control over smartphone use in classrooms was raised with the objective of protecting the educational context (Romanian Ministry of Education and Research, 2025). However, the present findings suggest that interventions should not focus exclusively on reducing technology use, but also on fostering balanced and self-regulated digital engagement. Specifically, school-based programs could focus heavily on digital literacy that helps adolescents reflect on the quality, not just the quantity, of their technology use, and develop an awareness of how digital habits affect their self-esteem and life satisfaction. It is all the more important that skills for maintaining meaningful offline activities alongside digital engagement are developed in schools. Such interventions supported by school psychologists or integrated into educational programs are likely to indirectly support self-esteem and life satisfaction and implicitly enhance flourishing levels.

## 5. Conclusions

The importance of this study lies in the fact that it shows the role of digital life balance in the psychological functioning of adolescents. The fact that digital life balance is related both directly and indirectly to flourishing via life satisfaction and self-esteem suggests that the way adolescents regulate their interaction with digital technologies is linked not only to their general evaluation of life but also to their sentiment of personal value. All of these factors contribute to optimal psychological functioning. The present findings highlight that digital life balance may be an important correlate of adolescent psychological functioning.

## Figures and Tables

**Table 1:** Demographic composition of the study sample.

Variables	*N* = 338	
Age	14–18 years	Mean age = 16.17, *SD* = 1.26
Sex	Boys	115 (34.0) *
	Girls	223 (66.0)
Residence	Urban	292 (86.4)
	Rural	46 (13.6)
Grade	IX–X	200 (59.2)
	XI–XII	138 (40.8)

*SD*—standard deviation; * percentages.

**Table 2:** Descriptive statistics of the analyzed variables.

Variables	M	*SD*	PR	OR	Skew.	Kurt.	ω
DLB	4.648	1.21	1–7	1.00–7.00	−0.118	−0.334	0.794
FL	5.302	0.97	1–7	1.63–7.00	−0.704	0.175	0.860
SE	2.860	0.60	1–4	1.20–3.90	−0.267	−0.555	0.867
LS	4.807	1.28	1–7	1.00–7.00	−0.379	−0.361	0.858

Note: DLB—digital life balance; FL—flourishing; SE—self-esteem; LS—life satisfaction; M—mean; *SD*—standard deviation; PR—possible range; OR—observed range; Skew.—skewness; Kurt.—kurtosis.

**Table 3:** Pearson correlation for study variables.

Variables	1	2	3
DLB	–		
FL	0.359 ***	–	
SE	0.295 ***	0.702 ***	–
LS	0.229 ***	0.722 ***	0.626 ***

Note: *** *p* < 0.001; DLB—digital life balance; FL—flourishing; SE—self-esteem scale; LS—life satisfaction.

**Table 4:** Evaluation indices of the model (reliability, convergent validity).

Variables	ρ_A_ (>0.70)	ρ_c_ (>0.70)	α (>0.70)	Loadings (Interval)	VIF (<3.30)	AVE (>0.50)
DLB	0.796	0.776	0.774	0.64–0.84	1.21–2.29	0.497
FL	0.883	0.876	0.861	0.34–0.84	1.43–2.33	0.475
SE	0.885	0.874	0.856	0.61–0.84	1.57–2.78	0.503
LS	0.881	0.868	0.867	0.62–0.91	1.65–2.69	0.573

Note: DLB—digital life balance; FL—flourishing; SE—self-esteem; LS—life satisfaction.

**Table 5:** Discriminant validity of the model: heterotrait–monotrait ratio of correlations (HTMT).

Constructs	1	2	3
1. DLB	–		
2. FL	0.432	–	
3. SE	0.352	0.834	–
4. LS	0.273	0.835	0.750

**Table 6:** Direct, indirect, and total effects in the structural model.

Direct Effect	β	95% CI	SErr	*t*-Value	*p*-Value <
DLB → FL	0.125	0.0382, 0.1958	0.03	3.157	0.001
DLB → SE	0.367	0.2490, 0.4810	0.05	6.237	0.001
DLB → LS	0.305	0.1946, 0.4218	0.05	5.295	0.001
SE → FL	0.456	0.3270, 0.5758	0.06	7.199	0.001
LS → FL	0.467	0.3512, 0.5964	0.06	7.495	0.001
Indirect effect					
DLB → FL	0.309	0.2197, 0.4064	0.04	6.622	0.001
Specific indirect effect					
DLB → SE → FL	0.166	0.1036, 0.2490	0.03	4.481	0.001
DLB → LS → FL	0.143	0.0857, 0.2160	0.03	4.311	0.001
Total effect					
DLB → FL	0.434	0.3236, 0.5405	0.05	7.729	0.001

Note: β—standardized path coefficient; SErr—standard error; *p*—probability value; significance threshold set at *p* < 0.001.

## Data Availability

Data are available in the Open Science Framework repository at https://osf.io/e7fm6/overview (accessed on 20 May 2026).

## Abbreviations

The following abbreviations are used in this manuscript:

ICT	Information and Communication Technology
DLB	Digital life balance
FL	Flourishing
LS	Life satisfaction
SE	Self-esteem
PLS-SEM	Partial Least Square-Structural Equation Modeling
